# Enhanced Antioxidant Activity under Biomimetic Settings of Ascorbic Acid Included in Halloysite Nanotubes

**DOI:** 10.3390/antiox8020030

**Published:** 2019-01-27

**Authors:** Andrea Baschieri, Riccardo Amorati, Tiziana Benelli, Laura Mazzocchetti, Emanuele D’Angelo, Luca Valgimigli

**Affiliations:** 1 Department of Chemistry “G. Ciamician”, University of Bologna, Via S. Giacomo 11, I-40126 Bologna, Italy; andrea.baschieri2@unibo.it (A.B.); riccardo.amorati@unibo.it (R.A.); 2 Department of Industrial Chemistry “Toso Montanari”, University of Bologna, Viale Risorgimento 4, I-40136 Bologna, Italy; tiziana.benelli@unibo.it (T.B.); laura.mazzocchetti@unibo.it (L.M.); emanuele.dangelo2@unibo.it (E.D.)

**Keywords:** halloysite, nanotube, nanoantioxidant, ascorbic acid, vitamin C, stability, antioxidant, peroxyl radicals, rate constant, biomimetic

## Abstract

Antioxidant activity of native vitamin C (ascorbic acid, AH_2_) is hampered by instability in solution. Selective loading of AH_2_ into the inner lumen of natural halloysite nanotubes (HNT) yields a composite nanoantioxidant (HNT/AH_2_), which was characterized and investigated for its reactivity with the persistent 1,1-diphenyl-2-picrylhydrazyl (DPPH•) radical and with transient peroxyl radicals in the inhibited autoxidation of organic substrates, both in organic solution (acetonitrile) and in buffered (pH 7.4) water in comparison with native AH_2_. HNT/AH_2_ showed excellent antioxidant performance being more effective than native ascorbic acid by 131% in acetonitrile and 290% (three-fold) in aqueous solution, under identical settings. Reaction with peroxyl radicals has a rate constant of 1.4 × 10^6^ M^−1^ s^−1^ and 5.1 × 10^4^ M^−1^ s^−1^, respectively, in buffered water (pH 7.4) and acetonitrile, at 30 °C. Results offer physical understanding of the factors governing HNT/AH_2_ reactivity. Improved performance of HNT/AH_2_ is unprecedented among forms of stabilized ascorbic acid and its relevance is discussed on kinetic grounds.

## 1. Introduction

L-Ascorbic acid (AH_2_) is, possibly, the most important water-soluble radical-trapping antioxidant in biological systems [1,2]. Unlike other animals, humans cannot biosynthesize it from glucose due to inactivation of the gene encoding the enzyme l-glucono-γ-lactone oxidase; therefore, ascorbic acid is an essential nutrient, known as vitamin C [3]. The recommended daily allowance (RDA) is 75 mg/day for women and 90 mg/day for man, however, its high-dose intake is considered safe up to 2000 mg/day [4,5,6,7]. Following isolation in 1928, its fundamental biological function became progressively clear, starting from the prevention of scurvy [8]. Ascorbic acid is essential to the biosynthesis of collagen and other biomolecules, and it also protects from several chronic diseases including neurodegenerative conditions, heart disease, eye disease, and can serve as an adjuvant in the treatment of cancer [8,9]. Emerging evidence highlights its role in regulating the epigenome [10]. Although its role is not limited to being an antioxidant, arguably its antioxidant activity and its redox properties are key to its overall biological function [1,2,3,4,5,6,11]. Water solubility is, however, not less important. For instance, it enables the repair (regeneration) of lipid-soluble tocopherol (vitamin E) from its phenoxyl radical at the water-lipid interface in biomembranes and lipoproteins, thereby “exporting the unpaired electron” from the lipid phase into the aqueous phase [12] and preventing so-called tocopherol-mediated peroxidation (TMP) [13].

Despite its importance as a biological antioxidant, the use of ascorbic acid for the protection of any kind of man-made material or for other biomedical applications, such as the use in cosmetic and dermatological formulations, has been abandoned due to its major instability [14,15]. Indeed, even the degradation (firstly oxidative, secondly hydrolytic) of naturally occurring vitamin C in fresh fruit and fruit juice upon storage is a current matter of major concern [16,17]. Due to its instability, the antioxidant activity of ascorbic acid can vary from excellent [18] to negligible [19], depending on the experimental conditions. To overcome this limitation a variety of derivatives of ascorbic acid have been proposed and have replaced it for different uses, although none appears as the ideal substitute. For instance, ascorbyl-2-glucoside [20] and sodium (or magnesium) ascorbyl phosphate [21] are obtained by blocking the OH group in position 2, thereby altering the redox properties of the molecule: while this approach guarantees higher oxidative stability and preserves water solubility, it also impairs the antioxidant activity, until enzymatic or spontaneous hydrolysis releases back the native ascorbic acid. As an alternative approach, esterification of ascorbic acid in position 6 (or 5) with lipophilic fatty acids, such as in ascorbyl palmitate or stearate maintains good antioxidant activity [22] while improving stability [23]; however, stabilization relies on transferring the molecule from the aqueous phase—where it can dissociate to ascorbate (p*K*_a_ = 4.17) becoming more reactive—to the lipid phase, by making it lipophilic, hence, profoundly altering its role. Other proposals like ascorbyl tetraisopalmitate combine both approaches [24]: they guarantee higher stability but they also sum-up the limitations.

In the light of the above considerations, non-covalent stabilization of ascorbic acid by inclusion in inert nanocarriers appears as an interesting alternative.

Nano-encapsulation of ascorbic acid in liposomes [25] and in porous silica nanoparticles [26] has been reported, these last affording improved stability [26], although actual antioxidant activity of these hybrid materials was not investigated.

Halloysite (HNTs) is a natural nanotubular clay with typical length of 800 nm and external diameter of 80 nm, composed of aluminosilicate and expressing (acidic) siloxane groups on the outer surface and (basic) aluminol at the inner surface. The difference between inner and outer surface allows different selectivity for molecules to be absorbed or linked in either surface [27,28,29,30]. Antioxidants loading on inner lumen has previously been reported in the case of a diarylamine for rubber stabilization [30], and in the case of natural phenolic antioxidants such as curcumin, silibinin [31], resveratrol [32], and quercetin [33] to obtain controlled antioxidant delivery systems.

Being water dispersible and non-toxic [34], HNT appeared optimal for protecting ascorbic acid in the inner cavity, so to create a fully biocompatible nanoantioxidant. The aim of this investigation was to assess whether inclusion of unstable ascorbic acid can improve its modest antioxidant performance under biomimetic settings and open new possibilities for unleashing its potential as an antioxidant. Preparation and properties of HNT/AH_2_ will be presented along with a detailed investigation on its reactivity with alkylperoxyl radicals and DPPH•, both in organic medium and in buffered water solution, by testing the protection of standard oxidizable materials through controlled autoxidation studies [35,36,37,38]. Kinetic measurements will show, for the first time, improved antioxidant performance of HNT/AH_2_ compared to the native material, whose physical-chemical understanding highlights the advantages of the proposed approach and paves the way to the rational development of even better biomimetic antioxidant materials.

## 2. Materials and Methods

### 2.1. Chemicals and Reagents

Ascorbic acid, AIBN (2,2′-azobis (2-methylpropionitrile)), AAPH ((2,2′-azobis (2-methylpropionamidine) dihydrochloride), PMHC (2,2,5,7,8-pentamethyl-6-chromanol), DPPH• (2,2-diphenyl-1-picrylhydrazyl), Chelex^®^ 100 sodium form, cumene, styrene, and tetrahydrofuran (unstabilized) were commercially available (Sigma-Aldrich, Milan, Italy). Solvents were HPLC or spectrophotometry grade and were used without further purification. THF was distilled under vacuum and stored under argon at 5 °C; the content in hydroperoxides was < 50 ppm (μg g^–1^), as determined spectrophotometrically (262 nm) by detection of Ph_3_P=O after titration with triphenylphosphine (Ph_3_P) in *iso*-propanol. Cumene and styrene were percolated through silica and through activated alumina to remove traces of peroxides and were stored under nitrogen. AIBN was recrystallized from methanol and stored at −18 °C. Halloysite nanoclay was purchased from Sigma-Aldrich: it had an average tube diameter of 70 nm and inner lumen diameter of 15 nm. Typical specific surface area of this halloysite is 65 m^2^ g^−1^, pore volume of ~1.25 cm^3^ g^−1^, refractive index 1.54, and specific gravity 2.53 g cm^−3^.

### 2.2. Buffer Preparation

Buffers were freshly prepared with bidistilled water and were stored in a refrigerator. The pH was adjusted with HCl or NaOH and checked by a glass-electrode pH meter (±0.05). Buffer pH 7.4: Na_2_HPO_4_ (0.595 g, 0.096 M) and NaH_2_PO_4_∙2H_2_O (0.125 g, 0.016 M) were dissolved in water (50 mL). Traces of transition metal ions were removed by treatment with Chelex^®^ 100–200 mesh particle size (5 g) for every 100 mL of sample, stirring gently for eight hours, then filtering the sample from the resin. Buffer solutions were mixed with the desired amount of THF (typically 3:1 by volume).

### 2.3. Preparation of Ascorbic Acid Modified Halloysite (HNT/AH_2_)

A dispersion of HNTs (500 mg, dry powder) in methanol (10 mL) was treated with ultrasound for 10 min to separate the tubes, after that 15 mL of ascorbic acid solution 0.19 M in methanol was added. The suspension was evacuated for 10 min to remove air from within the tubes, and then was cycled back to atmospheric pressure. This process was repeated three times in order to increase the loading efficiency. The obtained dispersions were magnetically stirred overnight at room temperature under Argon. The solid was separated by centrifugation, washed with methanol to remove any organic material adsorbed in the outer HNT surface (without applying vacuum), and dried under high vacuum for 5 h. The loading was repeated, in the same way, other two times by using the previously loaded HNTs and 15 mL of a freshly prepared solution of ascorbic acid 0.19 M in methanol. The dried solid of ascorbic acid modified halloysite (HNT/AH_2_) was investigated by means of TGA analysis to estimate the antioxidant loading into the HNT lumen.

### 2.4. Preparation of Ascorbic Acid Homogeneous Mixtures (M−x:AH_2_ + HNT)

Homogeneous mixtures ascorbic acid/halloysite (M-x:AH_2_ + HNT) were obtained by mixing a weighted amount of AH_2_ and HNTs in a mortar and pestle. Two M-x:AH_2_ + HNT at 4.4 wt% (M-4.4: AH_2_ + HNT) and 1.1 wt% (M−1.0: AH_2_+HNT) were prepared.

### 2.5. Thermogravimetric Analysis (TGA)

TGA experiments were carried out using a TA Instruments SDT-Q600 (New Castle, Germany) instrument on 12–18 mg samples in alumina crucibles. Samples were first heated from room temperature (RT) to 130 °C under inert atmosphere (nitrogen flow rate 100 mL/min) at a heating rate of 40° C/min, and isothermally kept at 130 °C for 40 min. Then, after switching to oxidizing atmosphere (air flow rate 100 mL/min), a second heating from 130 °C to 800 °C at a heating rate of 10 °C/min was carried out. The TGA of ascorbic acid sample was carried out under an oxidizing atmosphere with a single run from RT to 600 °C at a heating rate of 10 °C/min. Evaluation of the actual AA content in the samples was carried out according to Equation (1):

(1) $${\mathrm{AA}\%}_{\mathrm{wt}}=\frac{\left({\mathrm{WL}}_{130–800}-{\mathrm{WL}}_{HNT130–800}\right)}{\left(\mathrm{SW}-{\mathrm{WL}}_{25–130}\right)}\times 100$$

where WL_130–800_ is the weight loss of the AA containing samples in the second heating step from 130 to 800 °C after moisture removal, WL_HNT 130–800_ is the weight loss of the pristine reference HNT sample in the second heating step from 130 to 800 °C after moisture removal, SW is the starting weight of the sample, and WL_25–130_ is the moisture content evaluated during the first heating and isotherm step.

### 2.6. Dynamic Light Scattering

The hydrodynamic radii of dispersed particles were determined by dynamic light scattering (DLS) using a Malvern Zetasizer Nano ZS equipped with a 173° backscatter detector and a He-Ne laser (633 nm). The ACN or aqueous solutions (0.5 mg/mL) were placed into poly(styrene) cuvettes with four optical faces and 1 cm optical path. The solutions were prepared and filtered (0.45 μm filter) before the analysis. The field-time autocorrelation functions were analyzed by the inverse Laplace transform (ILT), which provides the decay rates (Г) of the diffusive modes. For the translational motion, the collective diffusion coefficient at a given concentration is D_t_ = Г/q^2^ where q is the scattering vector given by 4 пnλ^–1^ sin(θ/2) being *n* the acetonitrile refractive index, *λ* the wavelength (632.8 nm) and *θ* the scattering angle (173°). The hydrodynamic diameter (D_h_) was calculated by means of Stokes-Einstein relation.

### 2.7. UV-Vis Spectroscopy

UV-VIS spectra were recorded in a Jasco V550 double-beam spectrometer (Lecco, Italy) versus the corresponding solvent. All spectra were recorded at room temperature with bandwidth of 1 nm. The solutions were placed in standard 3.5 mL quartz cuvettes with path length of 10 mm.

### 2.8. Release of AH_2_ from HNT/AH_2_

Sample HNT/AH_2_ was dispersed in 10 mL of acetonitrile or in 3 mL of aqueous buffer (pH = 7.4), sonicated 1 min, stirred 24 min and centrifuged 5 min to minimize light scattering by HNT. The calibration lines were obtained adding different amount (50 to 300 μL) of ascorbic acid 1.42 mM to 3 mL of acetonitrile or ascorbic acid 1.65 mM to 3 mL of water buffer pH = 7.4.

### 2.9. Stability Studies of AH_2_

Ascorbic acid (3 mg) was dissolved in 10 mL of solvent (acetonitrile, methanol or aqueous buffer pH = 7.4). 200 μL were diluted in 3 mL of the same solvent to obtain a final concentration 1.1 × 10^–4^ M. Degradation was monitored spectrophotometrically at: *λ* = 238 nm for acetonitrile, *λ* = 246 nm for methanol and λ = 265 nm for aqueous buffer.

### 2.10. Determination of DPPH• Scavenging

The reaction mixtures were prepared by adding to a solution 143 μΜ of DPPH• radical in acetonitrile (3.5 mL), 50 μL of ascorbic acid 4 mM, or 1 mg of HNT/AH_2_. The solution was sonicated for 20 min and centrifuged for 10 min to minimize light scattering by HNT. Absorption of DPPH• was determined by recording the spectra in sequence until the absorbance was unchanged (8 h) and against a blank solution that contained only acetonitrile (λ_max_ = 517 nm, ε = 11,000 ± 50) [37].

### 2.11. Autoxidation Experiments

The kinetics of reaction with alkylperoxyl radicals was studied by autoxidation experiments in a differential oxygen-uptake apparatus based on a Validyne DP 15 pressure transducer built in our laboratory and described previously [39,40,41,42]. Autoxidation experiments consisted in monitoring the inhibition of oxygen consumption during the autoxidation of cumene (1.8 M) in acetonitrile or THF (3.1 M) in aqueous phosphate buffer. In a typical experiment, a thermal initiator was added to an air-saturated mixture of the oxidizable substrate and the solvent, i.e., AIBN (0.05 M) was used to start the autoxidation of cumene/acetonitrile 1:3 (*v/v*), and AAPH (25 mM) was the initiator for THF/water 1:3 (*v/v*) containing the buffer (0.1 M). Upon connection to the oxygen-uptake apparatus, the reacting mixture (sample) was equilibrated with an identical reference solution containing an excess of 2,2,5,7,8-pentamethyl-6-chromanol (to cause complete inhibition of the reference). When a constant rate O_2_ consumption was reached, 10–50 μL of a concentrated solution of the test antioxidant (AH_2_ or HNT/AH_2_) in the appropriate solvent (acetonitrile or water) was injected into the sample flask and oxygen consumption in the sample was measured. From the slope of oxygen consumption in the absence of antioxidant (−d[O_2_]/d*t*)_0_ = *R*_ox0_) and during the inhibited period (−d[O_2_]/d*t*) = *R*_ox_), *k*_inh_ values were obtained by using Equation (2) while the *n* coefficients were determined from the length of the inhibited period (τ) by using Equation (3), from the known rate of radical production by AIBN or AAPH (initiation rate, *R_i_*) [22]:

(2) $$\frac{R_{ox0}}{R_{ox}}-\frac{R_{ox}}{R_{ox0}}=\frac{nk_{inh}{\left[AH\right]}_{0}}{\sqrt{2k_{t}R_{i}}},$$

(3) $$n=\frac{R_{i}\tau }{\left[antioxidant\right]},$$

The 2*k_t_* values of cumene, styrene, and THF at 303 K are 4.6 × 10^4^, 4.2 × 10^7^, and 6.6 × 10^7^ M^−1^ s^−1^, respectively. The value of *R_i_* was determined under each experimental condition by using PMHC (2,2,5,7,8-pentamethyl-6-chromanol) or Trolox ((±)-6-hydroxy-2,5,7,8-tetramethylchromane-2-carboxylic acid) with *n* = 2 as a reference antioxidant: system = acetonitrile/cumene, *R*_i_ = 5.2 × 10^−9^ M s^−1^; system = acetonitrile/styrene, *R*_i_ = 6.7 × 10^−^^9^ M s^−^^1^; system = buffer pH 7.4/THF, *R*_i_ = 7.3 × 10^−9^ M s^−1^.

## 3. Results and Discussion

### 3.1. Preparation and Characterization of HNT/AH_2_

**Scheme 1:**
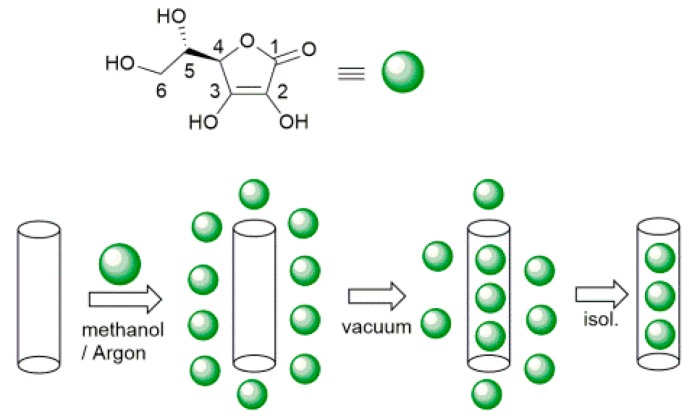
Preparation of ascorbic acid loaded halloysite nanotubes (HNT/AH_2_).

**Figure 1:**
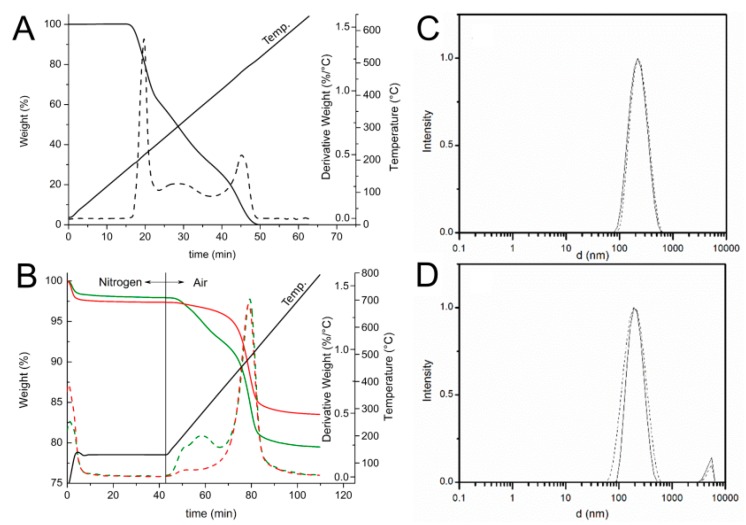
(**A**,**B**) TGA (solid line) and derivative DTG (broken line) thermograms of: (**A**) ascorbic acid (AH_2_) under air atmosphere at heating rate of 10 °C/min; and (**B**) HNT (▬▬) and HNT/AH_2_ (▬▬). The temperature profile applied during the measurement is reported. (**C**,**D**) DLS intensity distribution ofHNT (solid line) and HNT/AH_2_ (broken line) (**C**) in acetonitrile; and (**D**) in water solution (0.5 mg/mL).

Selective loading of ascorbic acid into HNT lumen was achieved by exploiting the different structure of the inner and outer surfaces of the nanotube with the basic inner lumen having much higher affinity for ascorbic acid (p*K*a = 4.2) than the moderately acidic outer silica surface [27]. Loading was carried out by repeated vacuum cycling of a HNT suspension in a solution of ascorbic acid in methanol, under argon atmosphere, followed by isolation of solid HNT/AH_2_ by centrifugation, washing of the outer surface with methanol (no vacuum applied) to remove AH_2_ adsorbed on the outer HNT surface, and drying under vacuum (Scheme 1). For optimal loading the whole cycle was repeated three times (additional cycles can be used to further increase loading). The composite solid HNT/AH_2_ was characterized by thermogravimetric analysis (TGA) with the aim to assess the real content of the antioxidant within the modified halloysite, as reported in the recent literature for similar products [33,43,44,45]. HNT/AH_2_ was analyzed by TGA, together with the unmodified sample (HNT) and the pristine AH_2_, as a reference. The TGA thermograms of pristine AH_2_ display a complex multi-step degradation pattern that, however, takes place only after reaching 190 °C (Figure 1A). Since halloysite tends to adsorb water, whose actual amount depends on the storage condition, each sample was preliminarily heated under nitrogen atmosphere from room temperature up to 130 °C and held at this temperature for 40 min. In these conditions, the adsorbed water is completely removed, thus allowing the direct comparison of any further weight loss in the samples (Figure 1B).

In these conditions, further undesired oxidation of the organic compounds can be safely ruled out, as demonstrated by the thermogravimetric curve of the pristine AH_2_ (Figure 1A) that does not show any weight loss at temperatures below 190 °C. Then, upon switching the atmosphere to air and raising the temperature up to 800 °C the degradation of all the organic and structural components occurs. As shown in Figure 1B, the HNT thermogram displays two weight losses: the first one around 100 °C (1–2%wt, 0–10 min) ascribed to the evaporation of water absorbed on the surface of the sample and the second one (13.8%wt), at about 500 °C (70–90 min), related to the loss of constitutional water [44,45]. The HNT/AH_2_ shows, besides the previously discussed weight losses, an additional degradation step, located in the 180–400 °C temperature range (40–70 min., partially overlapping the subsequent weight loss), which covers not only the structural water release, but also the ascorbic acid degradation, in agreement with the previously discussed TGA profile of pristine AH_2_ (Figure 1A).

In order to validate the applied method and verify the accuracy of the developed TGA approach, two homogeneous physical mixtures of known composition (1.0 wt% and 4.4 wt% mixtures of ascorbic acid in halloysite: M-1.0:AH_2_ + NHT and M-4.4:AH_2_ + NHT) were also analyzed in the same conditions. Results reported in Figure S1 (see Supplementary Materials) and Table 1 display that the preliminary water removal step does not affect the overall measurements and the applied method is sensitive enough to discriminate the AH_2_ content down to at least 1% wt.

Thus, by comparing the thermograms of HNT/AH_2_ with that one of the pristine HNT, once the absorbed water contribution has been removed, it is possible to evaluate the antioxidant fraction inside the inorganic frame, which results 4.6 ± 0.1 wt% in HNT/AH_2_.

The dimension of HNT/AH_2_ and the effect of the addition of ascorbic acid to the nanotubes was assessed by dynamic light scattering (DLS) studies in water and ACN solutions. As reported in Figure 1C,D, both pristine HNT and composite HNT/AH_2_ display a uni-modal distribution of particles size, centered around 230 nm, which is not substantially affected by the solvent. The measured particle dimension well compares with literature data concerning similar structures thus confirming that no aggregation occurs in both the analyzed samples even upon introduction of AH_2_ in the nanotubes.

This is at variance with previous work with HNT, in which functionalization of the external surface (as opposed to loading in the inner lumen) caused clustering of the nanotubes held together by interaction of the organic material on the outer surface, resulting and the appearance of a second broad-sized population with higher hydro-dynamic diameter [33].

**Figure 2:**
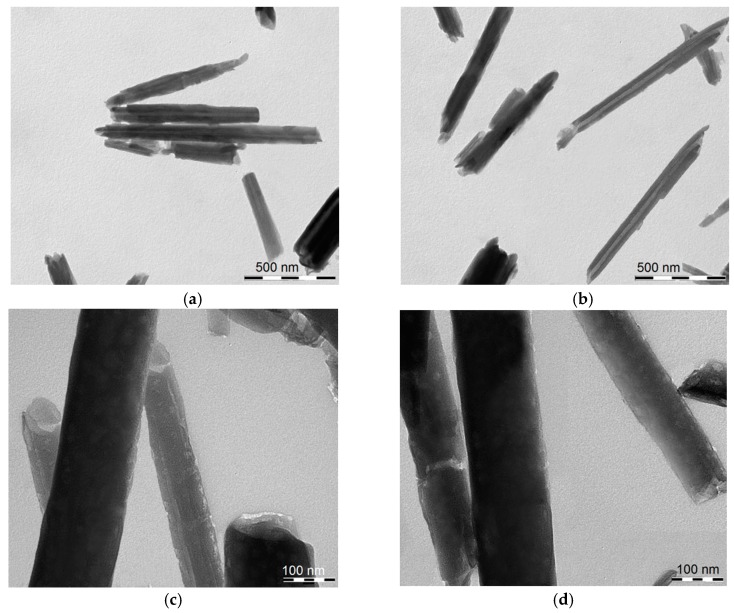
Transmission electronic microscopy (TEM) images of (**a**,**c**) pristine HNT; (**b**,**d**) of HNT/AH_2_ showing the nanotubular structure. Scale bars: (**a**,**b**) 500 nm; and (**c**,**d**) 100 nm.

The morphology of HNT/AH_2_ was imaged by TEM (Figure 2). It clearly appears that the tubular shape of the halloysite is maintained after AH_2_ loading, and no actual external modification is visible. The HNT diameter and length are in line with the typical values (see Section 1 and Section 2.1).

### 3.2. Release of Ascorbic Acid from HNT/AH_2_

The release of ascorbic acid from the HNT cavity was investigated both in water buffered at pH 7.4 and in acetonitrile chosen as the reference organic solvent, also used in subsequent measurements of antioxidant activity. Samples of HNT/AH_2_ were dispersed in the investigation solvent, briefly sonicated and the amount of released AH_2_ after 30 min at 298 K was evaluated by UV-Vis spectrophotometry upon calibration with pristine AH_2_. Results (see Supplementary Materials, Tables S1 and S2) indicate that only about half of the loaded AH_2_ is effectively released in 30 min, which is the typical time-lapse observed in inhibited autoxidation studies to assess antioxidant activity. On extending the analysis to eight hours, additional release was observed, however, the instability of ascorbic acid in the receptor solution prevented accurate quantitation. The more rapid release of a portion of ascorbic acid in solution, followed by a slower release on the remaining antioxidant, can be rationalized on the basis of previous work on quercetin-loaded HNT [33]. Considering the specific surface area [27] and nanotube size of halloysite (see Section 2.1) and that its cavity accounts for about 10% of its volume [46], and considering ascorbic acid molecular surface, it can be estimated that a monolayer of ascorbic acid adsorbed in the inner lumen would account for about 2 wt%, of the whole composite material, in line with previous calculations for the load of other molecules [33,46]. This estimate is close to 50% of the loaded ascorbic acid. Over such a monolayer, which is strongly bound to the inner surface, additional layers of more loosely-bound organic material stratify in the inner lumen, being more rapidly released upon interaction with the solvent [33], which accounts for the observed rapidly released 50% of load.

As anticipated instability of ascorbic acid in solution prevented accurate analysis of the full kinetics of release. To further investigate this aspect, the stability of pristine AH_2_ was monitored both in aqueous and organic solution.

### 3.3. Stability of Ascorbic Acid (AH_2_) in Solution

**Scheme 2:**
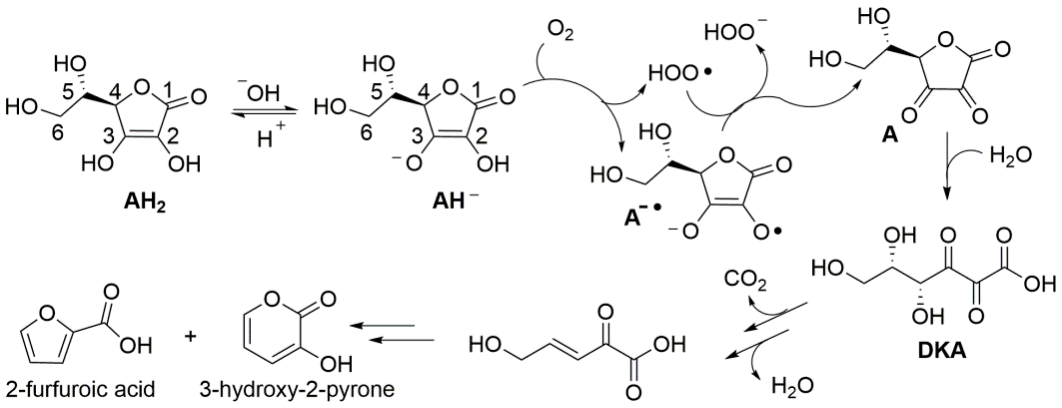
Aerobic degradation of ascorbic acid (AH_2_) in aqueous solution.

Despite the instability of ascorbic acid in solution has been known for almost a century, the kinetics and mechanism of degradation, along with the influence of experimental conditions are still debated and controversial. Product studies indicate that, under aerobic conditions in aqueous environment, degradation steps from formal two-electrons oxidation by molecular oxygen to yield dehydroascorbic acid (A) (Scheme 2), which hydrolyzes to diketogluconic acid (DKA) that further transforms in a variety of products [47], including furfuroic acid, 3-hydroxy-2-pyrone [14,47,48], and hydroxymethylfurfural [17]. Anaerobic (hydrolytic) degradation to furfural has also been observed [14]. Products’ distribution is reported to depend on pH [47]. Focusing on initial oxidation to dehydroascorbic acid (A), the process has been indicated to occur by a cascade of one-electron processes (an autoxidation chain-reaction) stepping either from AH_2_ or from the electron richer ascorbate mono-anion (AH^−^) [47], which would be prevalent at close to neutral pH (ascorbic acid has p*K*_a_ = 4.2) (Scheme 2). The chain reaction is mediated by the superoxide radical HOO•/O_2_^−^• (p*K*_a_ = 4.2) [49], as summarized in Scheme 2, and is catalyzed by traces of transition metal ions [50,51].

While some authors suggest that traces of metals are necessary to sustain the process [51], others indicate that oxidation can only occur from the mono-anion (AH^−^) [49]. Concerning the kinetics, while it is generally recognized as being first-order with respect of oxygen concentration [50], with respect of AH_2_ concentration it has been reported by some authors to be zero-order [16,50], by others to be first-order [17,49,51], yet by others second-order [52]. Similarly, Cabelli and Blelskl indicate a dependence on pH [49], while Wilson et al., reported that neither the mechanism nor the observed pseudo-first-order rate constant change significantly on changing the pH value [50]. This uncertainty is not totally surprising considering the complexity of the process; however, it does not aid the on-setting of strategies for the stabilization of ascorbic acid. Additionally, little is known on the stability of AH_2_ in organic solution.

**Figure 3:**
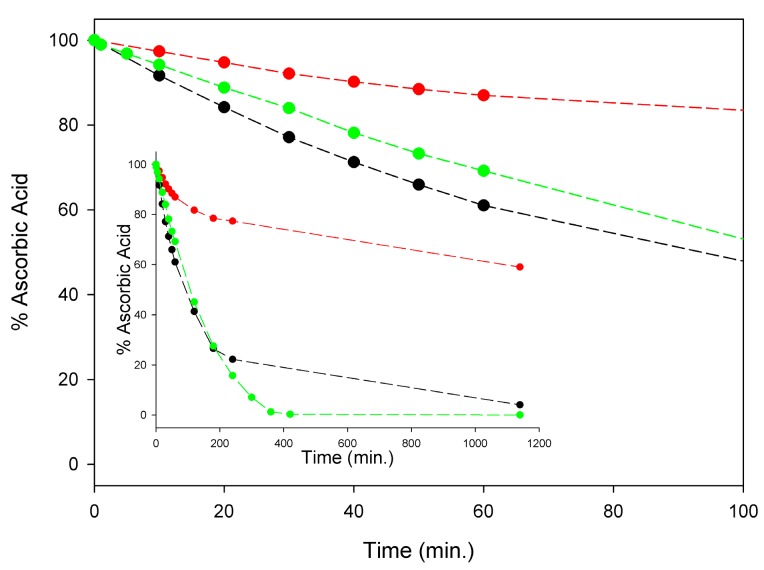
Stability of 100 µM ascorbic acid (AH_2_) in acetonitrile (red), water buffer pH = 7.4 (green) and methanol (black) at 298 K, in the presence of atmospheric oxygen. Insert shows the full time-course of AH_2_ decay expressed as remaining % of the starting concentration, while main graph represents the scale expansion for the first 100 min (kinetic analysis is shown in Figures S4–S6).

Although a detailed investigation is this regard was outside the scope of this study, in order to set our subsequent measurements of antioxidant activity on solid ground, we comparatively monitored the kinetics of degradation of AH_2_ under our experimental settings, i.e., in air-saturated solution at 298 K, in buffered (pH = 7.4) water, in methanol, and in acetonitrile. As summarized in Figure 3, significant degradation of AH_2_ was recorded in all the investigated solvents with methanol largely paralleling buffered water, while it was slower in acetonitrile. During the first four hours of monitoring, when about 80% of AH_2_ had disappeared in water or methanol (about 20% in acetonitrile), the process was found to follow pseudo-first-order kinetics (concentration of oxygen was kept constant), being of apparent first-order with respect to the AH_2_ concentration (see Figure S6). Apparent pseudo-first-order rate constants *k*_1st_ at 298 K were 1.18 × 10^−4^ s^−1^, 1.20 × 10^−4^ s^−1^, and 3.93 × 10^−5^ s^−1^, respectively, in air saturated water (pH = 7.4), methanol, and acetonitrile (see Supplementary Materials, Figures S4–S6). Non-zero-order dependence of the rate of degradation on AH_2_ concentration is important to the efficacy of our approach, as it relies on the slow release of AH_2_ in solution during the autoxidation, which is expected to slow down degradation by keeping a reduced concentration of AH_2_ in solution at any time.

### 3.4. Radical Trapping and Antioxidant Activity of HNT/AH_2_

#### 3.4.1. DPPH• Radical Trapping

In order to assess the radical reactivity or HNT/AH_2_ we monitored by spectrophotometry its reaction with persistent 2,2-diphenyl-1-pycryl-1-hydrazyl radical (DPPH•), which is often used as radical model [33,35]. Under matched experimental settings, in acetonitrile, at 298 K both HNT/AH_2_ and pristine AH_2_ rapidly reduced DPPH• to the corresponding hydrazine DPPH_2_. Pristine AH_2_ quenched DPPH• with a stoichiometric factor (the number of radicals trapped by one molecule of antioxidant) *n* = 1.9, in good agreement with the theoretical value of 2 (see Scheme S1) [35]. Therefore, by assuming identical stoichiometry of the reaction, we could determine the reducing equivalents of ascorbic acid released by HNT/AH_2_. Results (see Figure S7 in Supplementary Materials) indicated that HNT/AH_2_ releases 2.4 ± 0.1 wt% AH_2_ in the reactive reduced form, which is coincident, within experimental error, to the total value of AH_2_ released in solution (see Supplementary Materials, Tables S1 and S2). This implies that AH_2_ released is entirely in the reduced form and supports the idea that HNT/AH_2_ would act as an effective antioxidant material.

#### 3.4.2. Antioxidant Activity (ROO• Radical Trapping)

**Figure 4:**
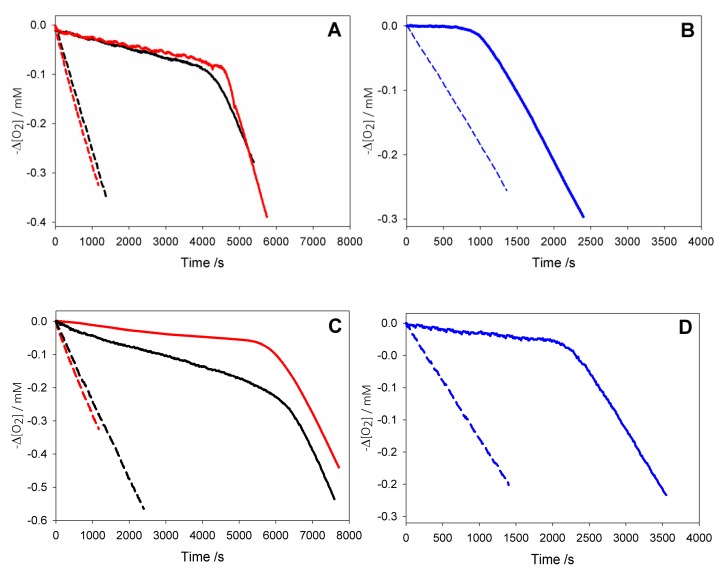
Oxygen consumption during the autoxidation of: (black) cumene (1.8 M) initiated by AIBN (0.05 M) at 30 °C in dry acetonitrile, or (red) with addition of 1% wt water (**A**,**C**), and (blue) during the autoxidation of THF (3.1 M) initiated by AAPH (25 mM) in phosphate buffer (0.1 M pH = 7.4) at 30 °C (**B**,**D**) without inhibitors (dash line); or in the presence of AH_2_ 2.5 × 10^−5^ M (solid line, **A**); or of AH_2_ 2.2 × 10^−5^ M (solid line, **B**); or in the presence of HNT/AH_2_ 0.18 mg/mL (corresponding to AH_2_ 2.5 × 10^−5^ M, solid line, **C**); or in the presence of HNT/AH_2_ 0.16 mg/mL (corresponding to AH_2_ 2.2 × 10^−5^ M, solid line, **D**).

Antioxidant activity of HNT/AH_2_ was evaluated in comparison with native AH_2_ (Figure 4), by measuring the rate constant (*k*_inh_) for the reaction with alkylperoxyl radicals (ROO•) during the controlled inhibited autoxidation of a standard substrate (Equations (4)–(9)) [11,13]. This is the most valuable method to gain quantitative information on antioxidant activity, as peroxyl radicals are responsible for the propagation step of peroxidation processes (Equation (6)) in most natural and man-made materials [35,36]. Studies were performed both in buffered (pH = 7.4) water solution (Figure 4B,D), using tetrahydrofuran as a standard substrate [18], and in acetonitrile, as a reference organic solvent, using cumene as the oxidizable substrate (Figure 4A,C) [22].

(4) $$Initiator\overset{R_{i}}{\to }R•,$$

(5) $$R•+O_{2}\overset{diffusion}{\to }ROO•,$$

(6) $$ROO•+RH\overset{k_{p}}{\to }R•+ROOH,$$

(7) $$2ROO•\overset{k_{t}}{\to }non\;radical\;products,$$

(8) $$ROO•+AH_{2}\overset{k_{inh}}{\to }ROOH+AH•,$$

(9) $$ROO•+AH•\overset{diffusion}{\to }ROOH+A,$$

(10) $$AH_{2}+O_{2}\to AH•+HOO•,$$

(11) $$AH•+O_{2}\to A+HOO•,$$

(12) $$AH_{2}{}^{}+HOO•\to AH•+HOOH,$$

(13) AH•+HOO•→A+HOOH,

(14) HOO•+RH→HOOH+R•,

Autoxidations were performed at 303 K using, respectively, 2,2′-azobis(2-amidinopropane) dihydrochloride (AAPH) or 2,2′-azobisisobutyronitrile (AIBN) as the initiator in aqueous or organic solution, and were followed by monitoring the oxygen consumption in a differential oxygen-uptake apparatus [36]. In the presence of antioxidants, oxidation of the substrate and oxygen consumption are slowed down and a neat inhibition period might become visible (as observed in Figure 4), until the antioxidant is consumed.

**Figure 5:**
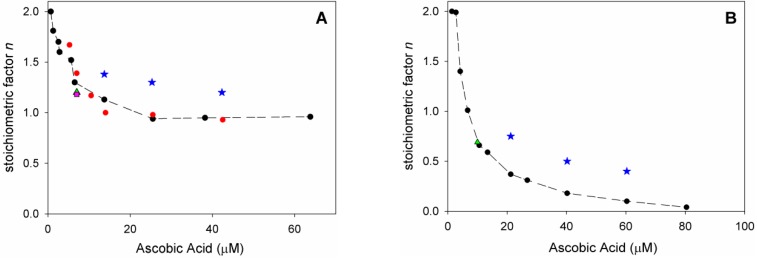
Variation of stoichiometric factor *n*: (**A**) during the autoxidation of Cumene (1.8 M) initiated by AIBN (0.05 M) in acetonitrile at 30° C (red), and upon addition of: 1% water (black), HNTs 0.25 mg/mL (green), 1% water and HNTs 0.25 mg/mL (pink) inhibited by variable amount of AH_2_ (7.0 × 10^−7^ M to 6.4 × 10^−5^ M, red, black, green, and pink), or inhibited by HNT/AH_2_ (blue); (**B**) during the autoxidation of THF (3.1 M) initiated by AAPH (25 mM) in phosphate buffer 0.1 M pH = 7.4 at 30 °C (black) and upon addition of HNTs 0.25 mg/mL (green), inhibited by variable amount of antioxidant AH_2_ (1.4 × 10^−6^ M to 8.1 × 10^−5^ M, black and green) or inhibited by HNT/AH_2_ (blue).

Analysis of O_2_ uptake affords both the rate constant *k*_inh_ and the stoichiometric factor *n*, respectively, from the slope and the length of the inhibition period in the plots, according to Equations (2) and (3). In acetonitrile, native ascorbic acid AH_2_ had fairly good antioxidant activity (Figure 4A) with measured *k*_inh_ of about 3 × 10^4^ M^–1^ s^–1^ which remains unchanged within experimental error upon addition of 1% water (see Table 2). Interestingly, however, its stoichiometric factor *n* varied with the concentration of the antioxidant: AH_2_ was able to trap two peroxyl radicals (Equations (8) and (9)) when it was used at very low concentration (lower than 1 µM); however, it progressively decreased to less than one peroxyl radical per molecule of AH_2_ when used at more realistic concentration of 25 µM or higher, as shown in Figure 5A. This progressive loss of antioxidant efficiency can be attributed to the competing oxidation of both ascorbic acid and the ascorbyl radical by molecular oxygen, with the generation of superoxide radicals (Equations (10) and (11)) that deplete the antioxidants (Equations (12) and (13)) and initiate new oxidative chains (Equation (14)). Indeed, the reaction of AH_2_ with oxygen causes both unproductive depletion of the antioxidant and pro-oxidant behaviour that partially competes with the antioxidant function. The importance of side reactions (Equations (10)–(14)), as compared to antioxidant reactions (Equations (8) and (9)), increases with the concentration of AH_2_ in solution, as it would affect the steady-state concentration of alkyperoxyl radicals, explaining the progressive decline in the antioxidant activity. As expected, in buffered water solution (pH 7.4) the antioxidant activity of ascorbic acid was much higher (see Figure 4B vs. Figure 4A), with *k*_inh_ as large as 1.7 × 10^6^ M^−1^ s^−1^, in line with previous studies [18] and with the notation that ascorbic acid has been selected by nature primarily as a water-soluble antioxidant. Higher reactivity with peroxyl radicals is due to preliminary acidic dissociation to ascorbate (the prevailing species at pH 7.4) which is electron-richer and rapidly reacts with peroxyl radicals by proton-coupled-electron-transfer (Equations (15) and (16)) [18]. Unfortunately, conversion into ascorbate may also facilitate the competing reaction with dioxygen (Equation (17)):

(15) $$AH_{2}+OH^{-}\to AH^{-}+H_{2}O,$$

(16) $$AH^{-}+ROO•\to A{•}^{-}+ROOH,$$

(17) $$AH^{-}+O_{2}\to A{•}^{-}+HOO•,$$

This is immediately evident upon comparing the length of the inhibited period in Figure 4B (in water) with those in Figure 4A (in acetonitrile) recorded at similar concentration of AH_2_. Indeed, in water the recorded loss of antioxidant efficiency upon increasing the concentration of AH_2_ was even more dramatic than in organic solution (Figure 5B) and, on increasing [AH_2_] from 1 µM to 81 µM, *n*-value dropped from about 2 to less than 0.1, confirming previous studies under similar experimental settings [19], and meaning almost complete abolition of the antioxidant role.

As expected, under similar settings either in acetonitrile or in buffered water, pristine HNT did not display any antioxidant behaviour; however, HNT/AH_2_ had excellent antioxidant activity (Figure 4C,D). Both in acetonitrile and in buffered water HNT/AH_2_ was tested at three different concentrations and afforded rate constants *k*_inh_ for the trapping of peroxyl radicals that were close to those recorded for pristine AH_2_. In dry acetonitrile *k*_inh_ was somewhat lower than that recorded for pristine AH_2_, possibly due to the too slow a release of AH_2_ in solution, and was also impaired by the modest solubility of ascorbic acid in the organic solvent. Indeed, upon addition of 1% water to the solvent to simulate “standard” acetonitrile and aid the release/solubilisation of AH_2_, the recorded *k*_inh_ value increased significantly (by about six-fold) surpassing the value recorded for pristine AH_2_ under the same settings (see also Table S4). In buffered water *k*_inh_ for HNT/AH_2_ was indistinguishable from that recorded for pristine AH_2_. More interestingly, however, compared to experiments with equivalent total amounts of AH_2_ in solution, HNT/AH_2_ had significantly higher stoichiometric factor both in acetonitrile and in water, being able to apparently trap more radicals per molecule of AH_2_ released in solution as can be seen from visual comparison of autoxidations inhibited by AH_2_ in solution (Figure 4A,B), or by equivalents amounts loaded into HNT (Figure 4C,D). Quantitative data are collected in Table 2 and in Table S4.

In acetonitrile the stoichiometric factor *n* was higher, on average, 131% compared to pristine AH_2_ (Figure 5A), while in buffered water the advantage of HNT/AH_2_ was even more striking, with higher *n* values being as much as 400% (on average 290%, three-fold) the value recorded for AH_2_ (Figure 5B). This excellent result is particularly important in buffered water (i.e., at close to physiological settings) as it completely subverts the scenario shown by AH_2_, caused by its competing reaction with oxygen (vide supra). In order to clarify the role of HNT on the antioxidant reactivity of AH_2_, parallel experiments were performed by adding, together, both pristine AH_2_ and pristine HNT in the autoxidising mixture. The mixture afforded antioxidant performance very similar to that of AH_2_ alone, and much lower than that recorded for composite HNT/AH_2_ (Table 2 and Table S4). This clearly indicates that the presence of halloysite nanotubes, per se, does alter the chemistry of ascorbic acid; therefore, the higher antioxidant performance of HNT/AH_2_ can entirely be attributed to the slower progressive release of AH_2_ in solution as it is consumed by quenching peroxyl radicals. This will maintain lower concentration of ascorbate at any time, slowing down its side reactions with dioxygen (Equations (10) and (17), thereby decreasing both the unproductive depletion of antioxidant and its competing pro-oxidant behaviour. This explanation is supported by the stability studies on pristine AH_2_ (vide supra) that showed first-order dependence of the rate of degradation on its concentration. Additionally, AH_2_ is stabilized from oxidation as long as it is in the HNT lumen.

It should be noted that improved antioxidant performance of composite or hybrid nanoantioxidants is a rather rare achievement and typically their antioxidant performance is lower, or similar to the corresponding native small-molecule antioxidant. For instance, some of us recently found that HNT carrying curcumin on the outer surface had significantly lower efficiency in trapping peroxyl radicals that native curcumin, both in polar and apolar solution [29]; similarly, it has been shown that both HNT grafted with Trolox on the outer surface and HNT loaded with quercetin in the inner lumen had lower efficiency in trapping both peroxyl radicals and DPPH• radical than native Trolox and quercetin, respectively [33]. Such findings are not limited to halloysite: for instance, it was shown that metal nanoparticles decorated with Trolox trapped peroxyl radicals with a stoichiometric factor about halved with respect to Trolox itself [53]; similarly, the DPPH• radical scavenging activity of SiO_2_ nanoparticles functionalized with gallic acid depended on particle size but was generally lower than that of native gallic acid [54]. On the other hand, self-assembled polymer/iron/tannic acid nanoparticles had antioxidant activity in vitro similar to native tannic acid [55]. There is no previous study in the literature comparing the direct antioxidant activity of ascorbic acid with that of any nano-sized composite, however, the DPPH• radical scavenging activity of SiO_2_/ascorbate nanoparticles were no better than that of ascorbate itself [26]. The decreased or not improved antioxidant performance of hybrid nanomaterials should not be read as diminishing their value, as they typically outperform the small-molecule antioxidants in other respects, such as controlled or targeted delivery. However, this scenario renders our current findings even more interesting.

The method used here to investigate HNT/AH_2_ represents a valuable tool to achieve physical understanding of the factors affecting the antioxidant behaviour of hybrid biomaterials by allowing their reaction with peroxyl radicals be studied on quantitative grounds.

Although HNT/AH_2_ does not reach the ideal stoichiometry of peroxyl radical trapping of *n* = 2, and there is still margin for improvement, our results demonstrate that protection of ascorbate into HNT lumen represents an unusually promising strategy, paving the way to the development of even more performing biomimetic antioxidant materials.

## 4. Conclusions

The good antioxidant behaviour of ascorbic acid either in organic or aqueous environment is largely hampered by its instability in solution, in the presence of atmospheric oxygen. Competitive oxidative degradation has apparent first-order dependence on the concentration of AH_2_ and is particularly remarkable under pseudo-physiologic conditions (pH 7.4 buffered water) where the stoichiometric factor for peroxyl radical trapping drops from two peroxyl radicals per molecule of AH_2_ to less than 0.1 on increasing the antioxidant concentration in the range 1–80 µM. Selective loading of AH_2_ in the inner lumen of HNT allows the creation of a composite nanomaterial that improves AH_2_ stability and makes HNT/AH_2_ able to slowly release AH_2_ in solution in the reduced form. HNT/AH_2_ proved to be a very effective antioxidant form of ascorbic acid, particularly suitable for applications in water solution under physiological settings where the antioxidant performance was enhanced on average by three-fold in the protection of a standard organic substrate. To the best of our knowledge, current results are unprecedented with respects to the various strategies aimed at improving the stability or the performance of ascorbic acid, including those based on its synthetic modification, and indicate that protection of AH_2_ in the lumen of inert nanotubes could be a most valuable strategy to develop optimized materials able to deliver the full properties of ascorbic acid in a stable and practical form.

## Figures and Tables

**Table 1:** TGA data of pristine (HNT) and modified (HNT/AH_2_) halloysite, along with plain ascorbic acid (AH_2_) and reference AH_2_ + HNT physical mixtures.

Samples	Moisture Content (wt%) ^1^	Weight Loss from 130 to 800 °C (wt%) ^2^	Estimated AH_2_ Fraction (%) ^3^
HNT	2.60 ± 0.05	13.80 ± 0.05	0
HNT/AH_2_	2.10 ± 0.05	18.40 ± 0.05	4.6 ± 0.1
M-1.0:AH_2_ + HNT	2.00 ± 0.05	15.00 ± 0.05	1.2 ± 0.1
M-4.4:AH_2_ + HNT	2.20 ± 0.05	18.20 ± 0.05	4.4 ± 0.1

^1^ Weight loss determined by TGA in nitrogen atmosphere (heating rate 10 °C/min) from RT to 130 °C and after an isotherm at 130 °C for 40 min. ^2^ Weight loss determined by TGA in air (heating rate 10 °C/min) from 130 to 800 °C, ^3^ Weight loss determined by TGA subtracting the HNT weight change in the range 130–800 °C (13.8%) from the AA modified HNT weight change in the range 130–800 °C.

**Table 2:** Antioxidant activity: rate constant for the reaction with ROO• radicals, *k*_inh_, measured in inhibited autoxidation experiments at 303 K (mean ± SD, *N* = 3). The number of radicals trapped by each antioxidant molecule, *n*, obtained from the same experiments, is shown in Figure 5 and reported numerically in the supporting information.

Sample	MeCN ^1^		MeCN + 1% Water ^1^		Buffer pH = 7.4 ^2^	
	*k*_inh_/M^−^^1^ s^−1^	*n*	*k*_inh_/M^−^^1^ s^−1^	*n*	*k*_inh_/M^−1^ s^−1^	*n*
HNT	No inhib	/	No inhib	/	No inhib	/
AH_2_	(2.5 ± 0.5) × 10^4^	Figure 5A	(3.1 ± 0.5) × 10^4^	Figure 5A	(1.7 ± 0.2) × 10^6^	Figure 5B
AH_2_ + HNT	(1.5 ± 0.3) × 10^4^	Figure 5A	(3.5 ± 0.5) × 10^4^	Figure 5A	(1.5 ± 0.2) × 10^6^	Figure 5B
HNT/AH_2_^3^	(8 ± 1) × 10^3^	Figure 5A ^4^	(5.1 ± 0.5) × 10^4^ ^5^	Figure 5A ^4^	(1.4 ± 0.3) × 10^6^	Figure 5B ^6^

^1^ Experiment performed with Cumene 1.8 M, AIBN 50 mM. ^2^ Experiment performed in phosphate buffer 0.1 M pH = 7.4, THF 3.1 M, AAPH 25 mM. ^3^ Apparent inhibition constant. ^4^ Experiments performed with [HNT/AH_2_] values of 0.095, 0.18 and 0.30 mg/mL, corresponding to [AH_2_] values of 1.4 × 10^−5^ M, 2.5 × 10^−5^ M and 4.2 × 10^−5^ M, respectively. ^5^ Experiment performed with Styrene 4.3 M, AIBN 50 mM. ^6^ Experiments performed with [HNT/AH_2_] values of 0.16, 0.31, and 0.46 mg/mL, corresponding to [AH_2_] values of 2.1 × 10^−5^ M, 4.0 × 10^−5^ M, and 6.0 × 10^−5^ M, respectively.

## Supplementary Materialss

The following are available online at www.mdpi.com/xxx/s1, Figure S1. Thermograms of HNT, HNT/AH_2_, and HNT + AH_2_ mixtures in air, Figure S2. Spectrophotometric analysis of AH_2_ release from HNT/AH_2_ in acetonitrile, Figure S3. Spectrophotometric analysis of AH_2_ release from HNT/AH_2_ in buffered water, Figure S4. Ascorbic acid decay in methanol at 25°, Figure S5. Ascorbic acid decay in buffered water at 25°, Figure S6. Ascorbic acid decay in acetonitrile at 25°, Figure S7. UV–VIS spectra of DPPH• reacting with AH_2_ and HNT/AH_2_, Table S1. Release of ascorbic acid (AH_2_) from HNT/AH_2_ in acetonitrile at 298 K, Table S2. Release of ascorbic acid (AH_2_) from HNT/AH_2_ in buffered water at 298 K, Table S3. Summary of AH_2_ release from HNT/AH_2_, Table S4: Stochiometric factors for peroxyl radical trapping by AH_2_ and HNT/AH_2_, Scheme S1. Reaction of ascorbic acid (AH_2_) with DPPH• radical.
