# Supplementary material for: Enhanced Antioxidant Activity under Biomimetic Settings of Ascorbic Acid Included in Halloysite Nanotubes
Source: Antioxidants (Basel). 2019 Jan 27;8(2):30. doi: 10.3390/antiox8020030 (PMC6406349; doi:10.3390/antiox8020030)
Supplement: Supplementary file 1 [file antioxidants-08-00030-s001.pdf]

Enhanced Antioxidant Activity under Biomimetic Settings of Ascorbic Acid included in Halloysite Nanotubes

*Andrea Baschieri,[1] Riccardo Amorati,[1] Tiziana Benelli,[2] Laura Mazzocchetti,[2] Emanuele D'Angelo,[2] Luca Valgimigli,[1,]**

[1]University of Bologna, Department of Chemistry "G. Ciamician", Via S. Giacomo 11, I-40126 Bologna, Italy.

[2]University of Bologna, Department of Industrial Chemistry "Toso Montanari", Viale Risorgimento 4, I-40136 Bologna, Italy.

*luca.valgimigli@unibo.it.

Supplmentary Material

Table of contents | *Page*

Figure S1: Thermograms of HNT, HNT/AH_2 and HNT + AH_2 mixtures in air 2
Table S1. Release of ascorbic acid (AH_2) from HNT/AH_2 in acetonitrile at 298 K 2
Table S2. Release of ascorbic acid (AH_2) from HNT/AH_2 in buffered water at 298 K 2
Table S3. Summary of AH_2 release from HNT/AH_2. 3
Table S4. Stoichiometric factors for peroxyl radical trapping by AH_2 and HNT/AH_2 3
Figure S2. Spectrophotometric analysis of AH_2 release from HNT/AH_2 in acetonitrile 4
Figure S3. Spectrophotometric analysis of AH_2 release from HNT/AH_2 in buffered water 5
Figure S4. Ascorbic acid decay in methanol at 25° 6
Figure S5. Ascorbic acid decay in buffered water at 25° 6
Figure S6. Ascorbic acid decay in acetonitrile at 25° 6
Figure S7. UV–vis spectra of DPPH• reacting with AH_2 and HNT/AH_2 7
Scheme S1. Reaction of ascorbic acid (AH_2) with DPPH• radical, 7
References 8

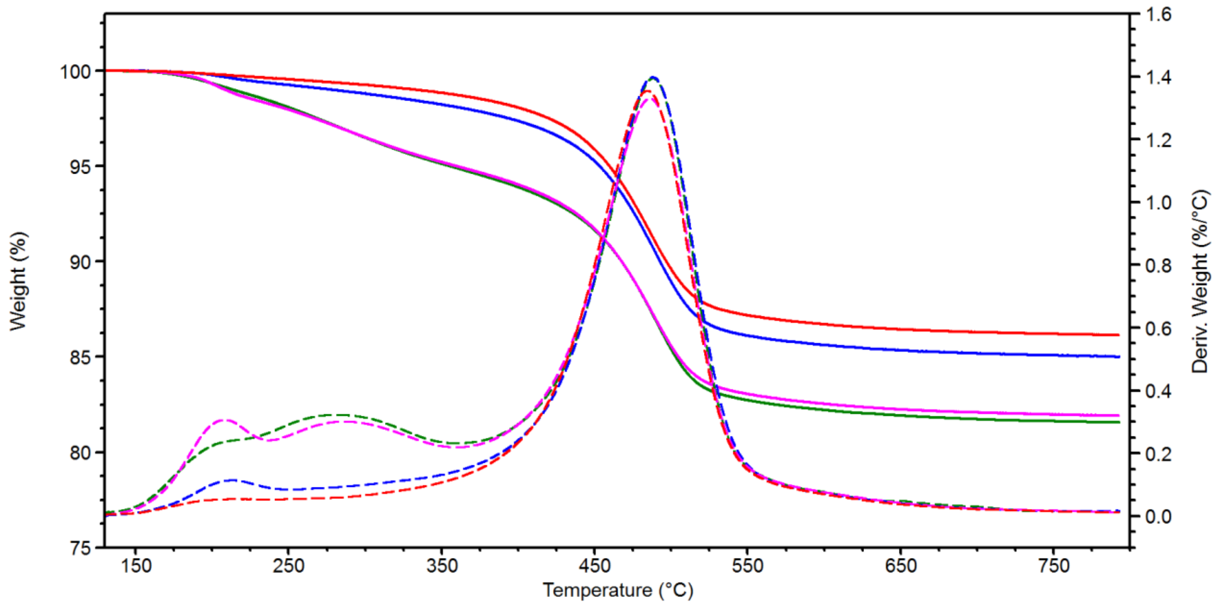

Figure S1. TGA thermograms of HNTs (▬▬), HNT/AH_2 (▬▬), M-1.0: AH_2+HNT (▬▬), M-4.4: AH_2+HNT (▬▬) and their first derivative curves (broken lines) under air atmosphere from 130 up to 800°C. The curves are cleared of the absorbed water contribute.

Table S1. Release of ascorbic acid (AH_2) from HNT/AH_2 expressed as mg in 3 mL of acetonitrile at 298 K (data correspond to experiments in Figure S2)

Entry	HNT/AH_2 (mg)	Abs.	AH_2 released (mg)	% AH_2 [a]
1	1.9	0.2327	0.0444	2.33
2	1.8	0.2159	0.0412	2.29
3	3.2	0.4484	0.0856	2.67
mean ± SD				2.5±0.2

[a] % Ascorbic acid released (w/w) from the weighted HNT/AH_2 sample

Table S2. Release of ascorbic acid (AH_2) from HNT/AH_2 expressed as mg in 3 mL of buffered (pH = 7.4) water at 298 K (data correspond to experiments in Figure S3)

Entry	HNT/AH_2 (mg)	Abs.	AH_2 released (mg)	% AH_2 [a]
1	1.0	0.6915	0.0228	2.3
2	1.9	1.4154	0.0460	2.4
3	4.0	2.7029	0.0878	2.2
mean ± SD				2.3±0.1

[a] % Ascorbic acid released (w/w) from the weighted HNT/AH_2 sample

Table S3. Summary of AH_2 release (mean ± SD) after 30 min, versus the amount loaded in HNT/AH_2, in buffered (pH 7.4) aqueous solution and in acetonitrile at 298K. Percentage refer to the weight of ascorbic acid over the weight of composite material HNT/AH_2

Solvent	AH_2 load in HNT/AH_2	AH_2 released (%)
Acetonitrile	4.6 %	2.5±0.2%
Water (pH 7.4)	4.4 %	2.3±0.1%

Table S4. Antioxidant activity: number of radicals trapped by each antioxidant molecule, *n*, at different concentration of the antioxidant AH_2 (in parenthesis), measured in inhibited autoxidation experiments at 303 K (mean ± SD, *N* = 3).

**Sample**	**MeCN**[a]	**MeCN + 1% water**[a]	**Buffer pH=7.4**[b]
	n	***n***	***n***
HNT	/	/	/
AH_2	1.0<br>($1.4x10^{-5}$M)	1.1<br>($1.4x10^{-5}$M)	0.4<br>($2.1x10^{-5}$M)
	1.0<br>($2.5x10^{-5}$M)	0.9<br>($2.5x10^{-5}$M)	0.2<br>($4.0x10^{-5}$M)
	0.9<br>($4.2x10^{-5}$M)	0.9<br>($3.8x10^{-5}$M)	0.1<br>($6.0x10^{-5}$M)
AH_2+HNT	1.2[c]<br>($7.0x10^{-6}$M)	1.2[c]<br>($7.0x10^{-6}$M)	0.7[c]<br>($1.0x10^{-5}$M)
HNT/AH_2	1.4<br>($1.4x10^{-5}$M)	1.4[d]<br>($1.4x10^{-5}$M)	0.8<br>($2.1x10^{-5}$M)
	1.3<br>($2.5x10^{-5}$M)	1.3 [d]<br>($2.5x10^{-5}$M)	0.5<br>($4.0x10^{-5}$M)
	1.2<br>($4.2x10^{-5}$M)	1.2 [d]<br>($4.2x10^{-5}$M)	0.4<br>($6.0x10^{-5}$M)

[a]Experiment performed with Cumene (1.8 M), AIBN (0.05 M). [b]Experiment performed in Phosphate Buffer 0.1 M pH = 7.4, THF 3.1 M, [AAPH] 25 mM. [c][HNT] = 0.25 mg/mL. [d]Experiment performed with Styrene (4.3 M), AIBN (0.05 M).

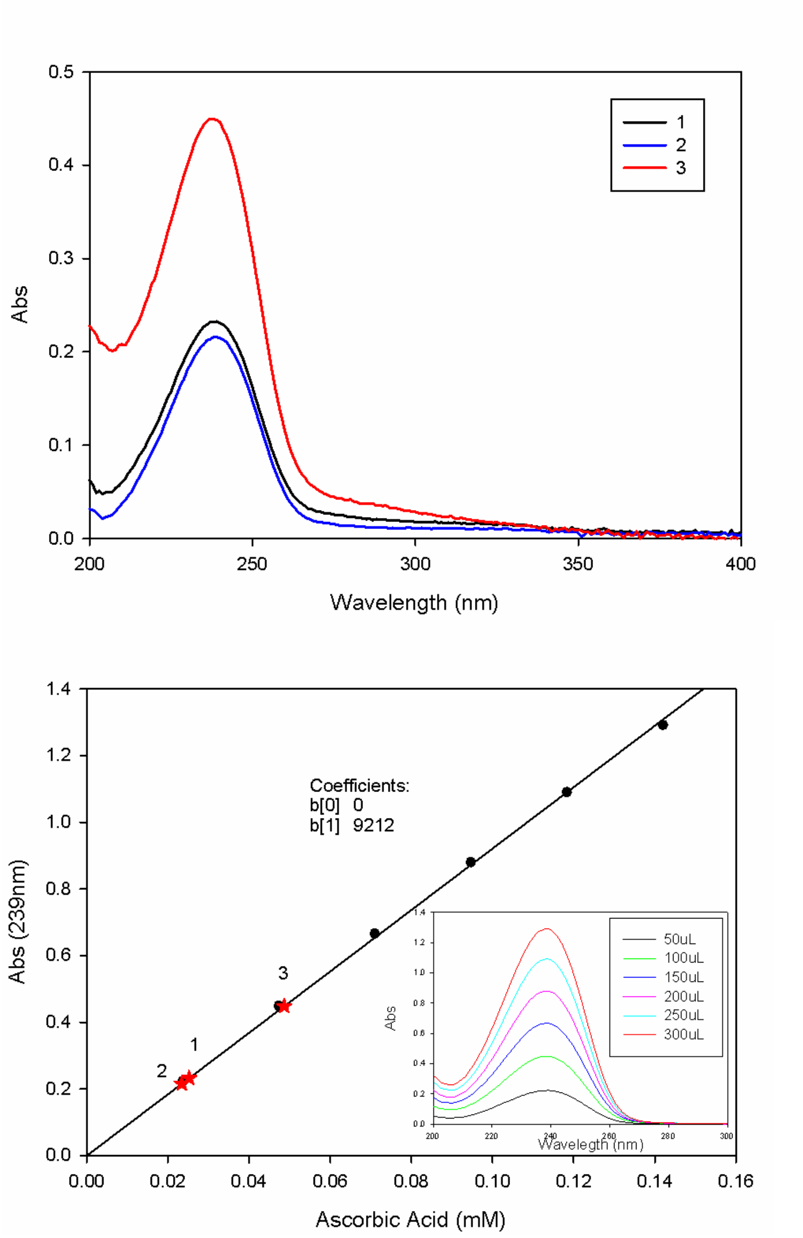

Figure S2. Spectrophotometric analysis of AH_2 release from samples of HNT/AH_2 in 3 mL acetonitrile, sonicated for 1 min., stirred for 24 min. and centrifuged for 5 min. to minimize light scattering due to HNT (top panel). The calibration line (lower panel, black circles) was obtained by addition of different volumes (reported in the insert in µL) of a stock solution of genuine AH_2 1.42 mM to 3 mL of acetonitrile. In lower panel experiments with HNT/AH_2 samples are shown as red stars. Numbering refers to table S1.

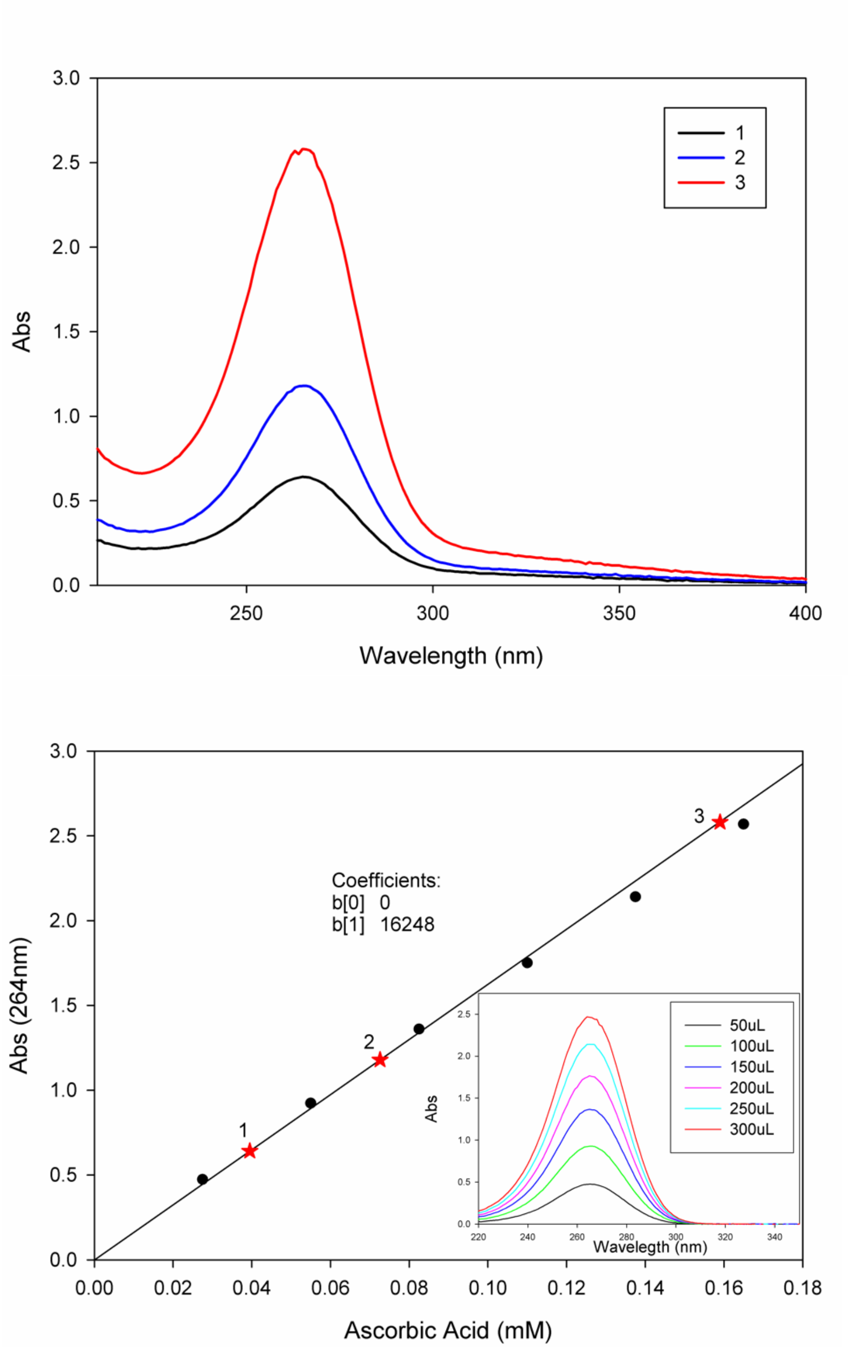

Figure S3. Spectrophotometric analysis of AH_2 release from samples of HNT/AH_2 in 3 mL aqueous buffer (pH = 7.4) sonicated 1 min., stirred 24 min and centrifuged 5 min. to minimize light scattering due to HNT (top panel). The calibration line (lower panel, black circles) was obtained by addition of different volumes (reported in the insert in µL) of a stock solution of genuine AH_2 1.65 mM to 3 mL of aqueous buffer. In lower panel experiments with HNT/AH_2 samples are shown as red stars. Numbering refers to table S2.

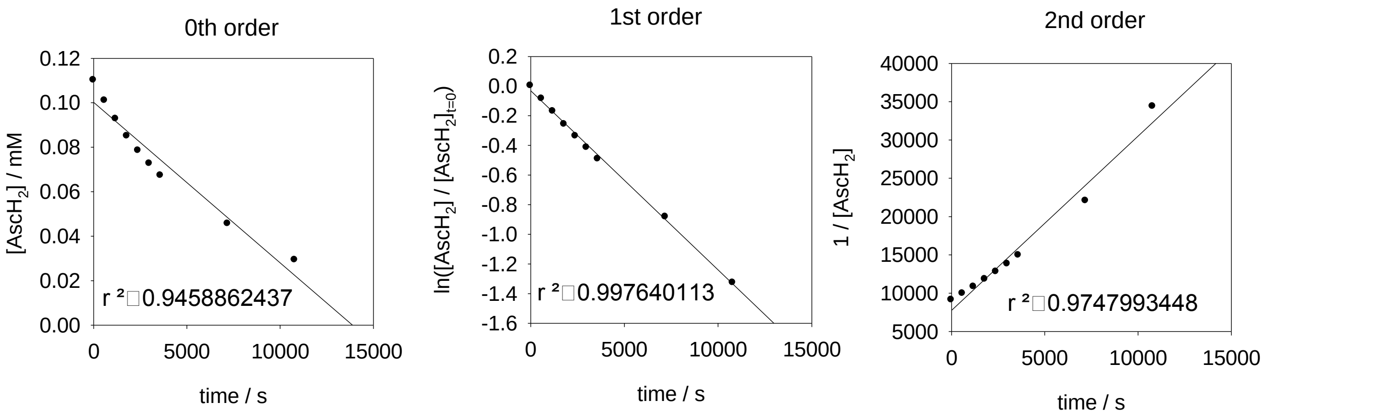

Figure S4. Ascorbic acid decay in methanol at 25°, analyzed to determine the reaction order. The best fit is obtained with the first order data analysis. The first order constant is 1.20×10^{-4} s^{-1}, which corresponds to a second-order rate constant of 0.06 $M^{-1}s^{-1}$ considering the solubility of oxygen in methanol (2.0 mM at 25°, 0.2 Atm[1]).

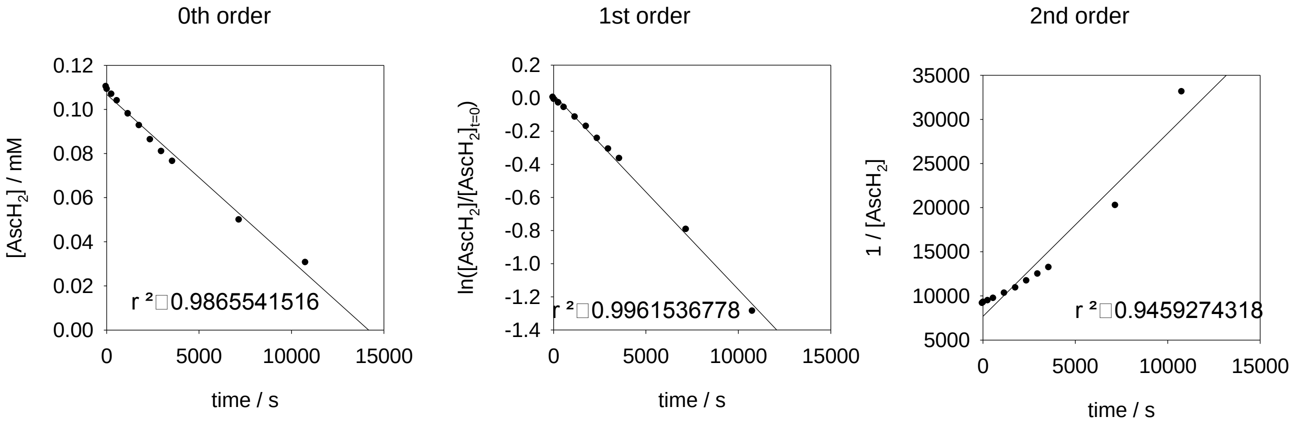

Figure S5. Ascorbic acid decay in water at 25°, analyzed to determine the reaction order. The best fit is obtained with the first order data analysis. The first order constant is 1.18×10^{-4} s^{-1}, which corresponds to a second-order rate constant of 0.56 $M^{-1}s^{-1}$ considering the solubility of oxygen in buffered water (0.21 mM at 25°, 0.2 Atm[2]).

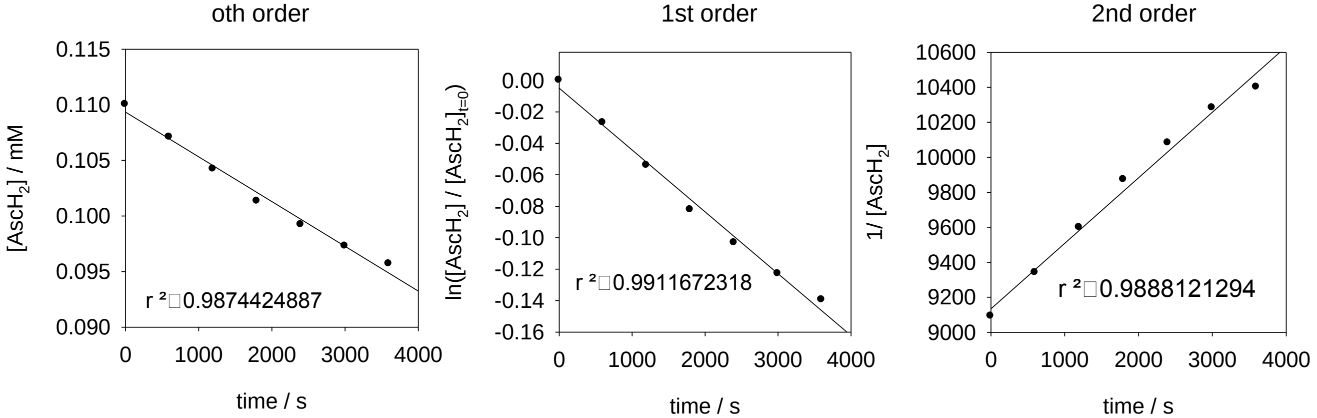

Figure S6. Ascorbic acid decay in acetonitrile at 25°, analyzed to determine the reaction order. The best fit is obtained with the first order data analysis. The first order constant is 3.93×10^{-5} s^{-1}, which corresponds to a second-order rate constant of 0.03 $M^{-1}s^{-1}$ considering the solubility of oxygen in acetonitrile (1.3 mM at 25°, 0.2 Atm[3]).

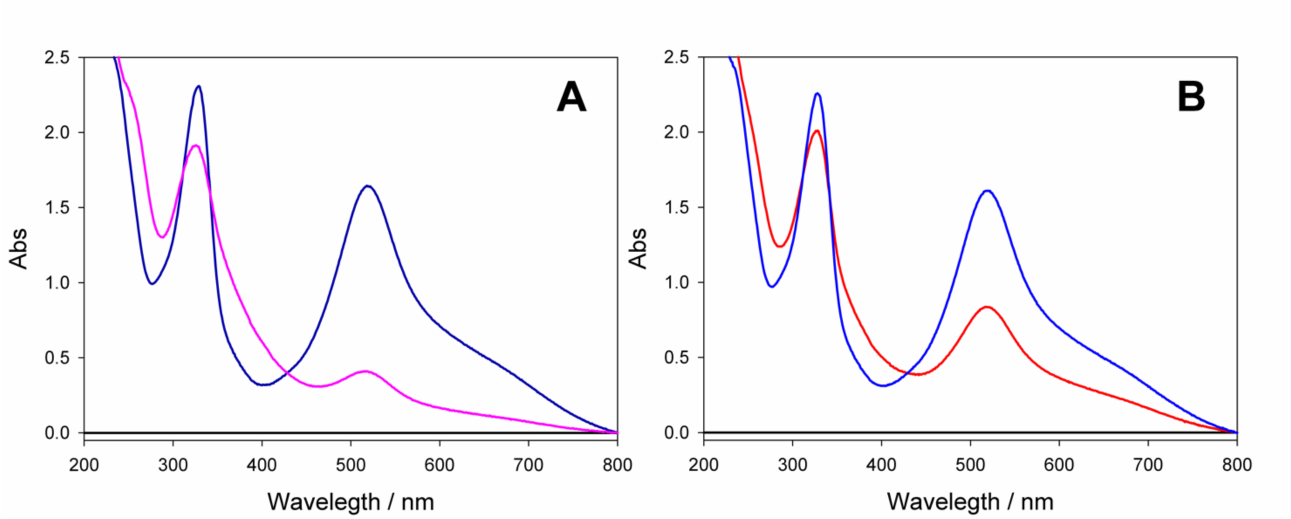

Figure S7. UV–vis (200–800 nm) absorption spectra of: (**A**) DPPH• 143 μM in acetonitrile (dark blue) and after addition of ascorbic acid 57 μM (pink), (**B**) DPPH• 143 μM in acetonitrile (blue) and after addition of HNT/AH_2 0.29 mg/mL (red).

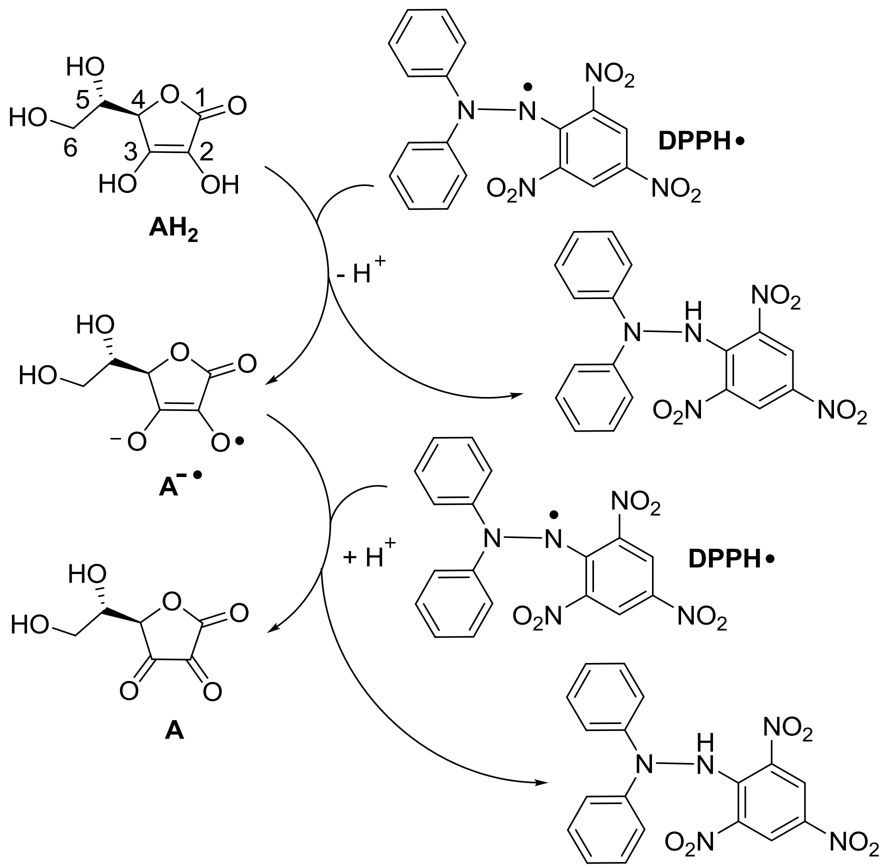

Scheme S1. Reaction of ascorbic acid (AH_2) with DPPH• radical, explaining the observed stoichiometry.

References

1) Sato, T.; Hamada, Y.; Sumikawa, M.; Araki, S.; Yamamoto, H. Solubility of Oxygen in Organic Solvents and Calculation of the Hansen Solubility Parameters of Oxygen, *Ind. Eng. Chem. Res.*, **2014**, *53*, 19331–19337.

2) https://www.engineeringtoolbox.com/oxygen-solubility-water-d_841.html.

3) Li, Q.; Batchelor-McAuley, C.; Lawrence, N. S.; Hartshorne, R. S.; Compton, R. G. Anomalous Solubility of Oxygen in Acetonitrile/Water Mixture Containing Tetra-n-butylammonium Perchlorate Supporting Electrolyte; the Solubility and Diffusion Coefficient of Oxygen in Anhydrous Acetonitrile and Aqueous Mixtures, *J. Electroanal. Chem.*, **2013**, *688*, 328-335.
